# Expression and clinical value of key m^6^A RNA modification regulators in tuberculosis

**DOI:** 10.3389/fimmu.2025.1729362

**Published:** 2026-01-14

**Authors:** Hongfei Du, Hang Wang, Yan Yang, Qiao Yu, Zhongyong Jiang, Ying Xu

**Affiliations:** 1 Department of Clinical Laboratory, The First Affiliated Hospital of Chengdu Medical College,School of Clinical Medicine,Chengdu Medical College, Chengdu, Sichuan, China; 2 School of Laboratory Medicine, Chengdu Medical College, Chengdu, Sichuan, China; 3 Department of Clinical Laboratory, The Affiliated Cancer Hospital of Chengdu Medical College, Chengdu Seventh People’s Hospital, Chengdu, Sichuan, China

**Keywords:** N6-methyladenosine, m6A RNA modification, tuberculosis, pulmonarytuberculosis, immune cell, immune process, immunemicroenvironment

## Abstract

**Background:**

N6-methyladenosine (m^6^A), the most prevalent and reversible post-transcriptional RNA modification, is involved in the progression of various diseases. Nonetheless, the role of m^6^A modification in Tuberculosis (TB) pathogenesis remains unknown. Here, we investigated the general expression patterns and potential functions of m^6^A regulators in TB.

**Methods:**

The differentially expressed m^6^A genes between the healthy and TB groups were evaluated using the public Gene Expression Omnibus (GEO) database, and quantitative real-time PCR (qRT-PCR) was used to test the expression of key m^6^A regulators in our collected human TB and healthy samples. Random forest and LASSO regression analysis were performed to determine the prognostic performance of m^6^A regulators in TB patients. The relationship between m^6^A regulators and immune cells and immune reaction activity was analyzed through single-sample gene set enrichment analysis (ssGSEA). Unsupervised clustering was used to confirm that m^6^A regulators induced m^6^A modification patterns. The relationship between m^6^A modification patterns and the immune microenvironment, biological function, and TB subtype construction was evaluated by using Gene Set Enrichment Analysis (GSEA), Gene Ontology (GO) analysis and KEGG pathway analysis.

**Results:**

Our data revealed seven differentially expressed m^6^A -related genes-METTL3, VIRMA, YTHDF1, YTHDC1, YTHDC2, ELAVL1and LRPPRC mRNA-confirmed as critical m^6^A regulators in TB. The excellent diagnostic significance of these genes was further supported by the random forest, LASSO regression and clinical samples, which achieved a high area under the ROC (0.97). Unsupervised clustering classified patients into two m^6^A patterns with different immune microenvironment and biological feature.

**Conclusions:**

Our study provides an overview of the expression patterns and potential roles of key m^6^A regulatory genes as diagnostic biomarkers and immunotherapy targets for TB, revealing their functions in TB pathogenesis. Our data may offer a valuable resource to guide both mechanistic and therapeutic analyses of key m^6^A regulators in TB.

## 1 Introduction

Tuberculosis (TB), caused by Mycobacterium tuberculosis (Mtb), is the most communicable infectious disease, with high morbidity and mortality (1). Despite continued efforts in treatment, TB remains a major public health crisis (2). However, only approximately 10% of patients infected with Mtb develop active TB, while approximately 90% of the infected cases exhibit latent infection, indicating a key role of host innate immunity in preventing Mtb infection (3). As reports demonstration, Macrophages, the first line of human host immunity in controlling Mtb infection, can act as different innate immune defenses against Mtb and clear foreign pathogenic microorganisms (4). However, the molecular mechanisms involved in the regulation of macrophage defense against Mtb infection have not been fully explored. Thus, it is important to understand TB pathogenesis of TB and to identify effective therapeutic targets.

In the term of RNA epigenetics, N6-methyladenosine (m^6^A) is the most prevalent chemical modification of eukaryotic mRNAs among different known RNA modifications, and it can mediate many biological processes of RNA, containing splicing, nuclear export, stability and translation efficiency (5–10). Several studies have demonstrated that internal m^6^A modifications play a role in regulating pathogen infection. For instance, m^6^A modification enhances the replication of enterovirus type 71 (EV71) (11). Conversely, m^6^A negatively mediates the production or release of infectious hepatitis C virus (HCV) viral particles (12). RNA m^6^A reader YTHDF1 regulate inflammation via enhancing NLRP3 translation (13). while the role of m^6^A RNA methylation regulators in TB and their correlation with TB genes remain poorly understood. A systematic understanding of m^6^A regulatory expression and genetic variation in TB heterogeneity will promote the validation of therapeutic targets based on RNA methylation. Therefore, in this study, we explored and validated the expression patterns and functions of m^6^A RNA methylation regulators in TB through public database and clinical samples.

## 2 Materials and methods

### 2.1 Data acquisition and analysis

**Figure 1:**
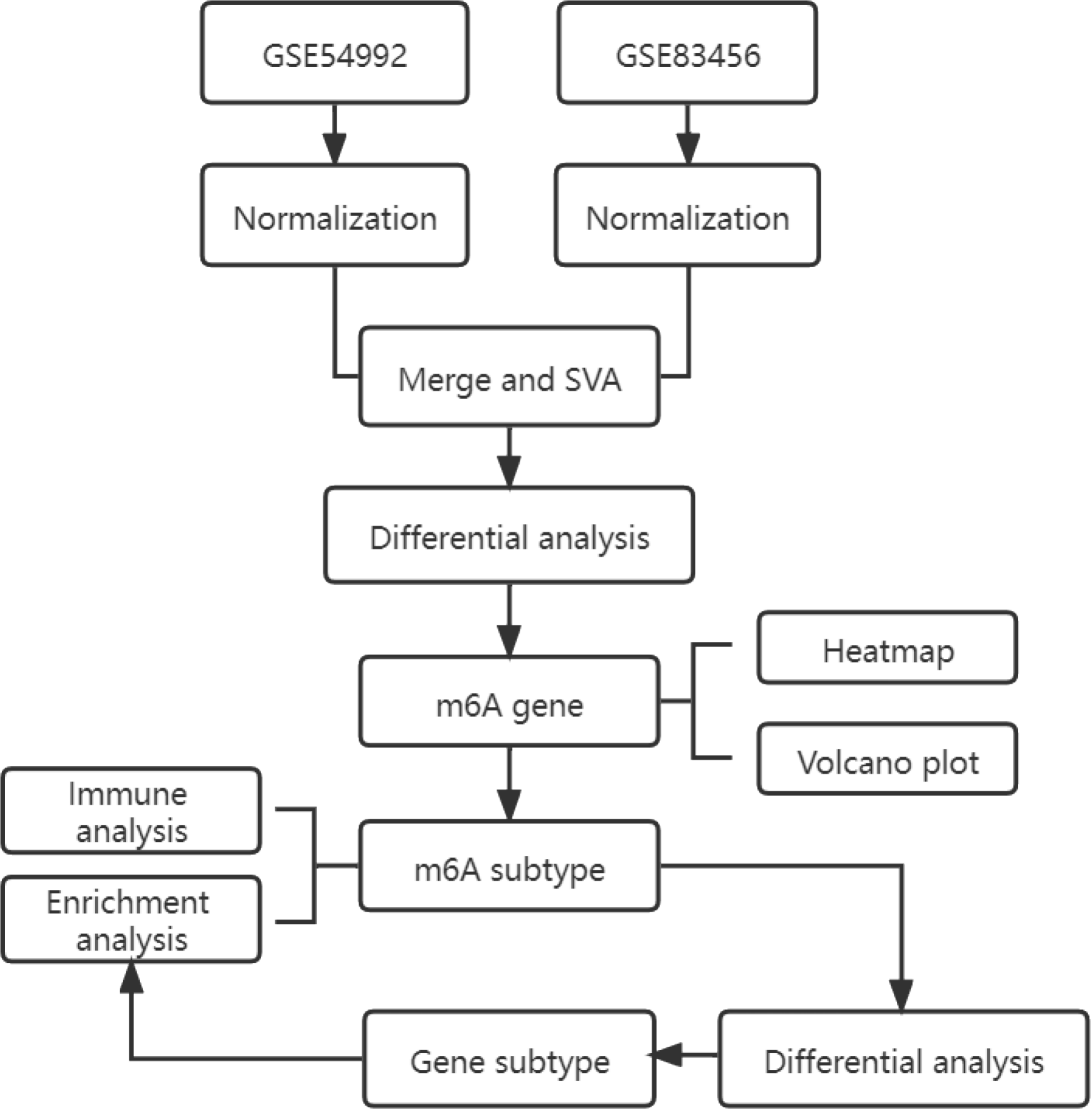
The overall chart of this study.

Two Homo sapiens gene array expression series matrix files of tuberculosis peripheral blood samples, including GSE54992 (14) and GSE83456 (15), were collected from GEO (**Table 1**). GSE54992, in which tests were performed on the Affymetrix Human Genome U133 Plus 2.0 Array (GPL570 platform, Affymetrix, Inc.), contains the expression profiles of 39 samples, which comprise 27 active pulmonary TB cases, six healthy controls, and six latent TB cases. A total of 33 TB cases and healthy controls were enrolled, and 6 samples of latent TB were excluded from our investigation. GSE83456(GPL10558) contains 45 humans with pulmonary TB, 47 humans with extra-pulmonary TB, 49 cases of pulmonary sarcoidosis, and 61 healthy human controls. A total of 106 patients with PTB and healthy controls were recruited for this study. The flowchart of the study is displayed in **Figure 1**. The m^6^A differentially expressed genes (DEGs) analysis between pulmonary TB and healthy controls were conducted by using “limma”R packages (version 3.64.1) (16). A volcano plot was used to visualize the expression of DEGs through “pheatmap”R packages (version 1.0.13) (17) and a heatmap diagram was performed to exhibit the expression of m^6^A regulators in TB and normal control by “ggplot” R packages (version 3.5.2) (18).

**Table 1:** The information of dataset.

Dataset	Normal	Disease	Platform	Organism	Tissue	Reference
GSE54992	6	27	GPL570	Homo sapiens	Blood	(14)
GSE83456	61	45	GPL10558	Homo sapiens	Blood	(15)

### 2.2 Identification of m^6^A RNA methylation regulatory genes in TB

Twenty-two widely studied m^6^A methylation regulators containing Eraser: FTO, ALKBH5; Writer: VIRMA, WTAP, ZC3H13, METTL14, METTL3, CBLL1, RBM15, RBM15B; Reader: YTHDF1, YTHDF2, YTHDF3, YTHDC1, YTHDC2, HNRNPC, HNRNPA2BP1, IGF2BP1, IGF2BP2, IGF2BP3, ELAVL1, LRPPRC were confirmed from published reports. Spearman correlation analysis was conducted to evaluate the association between m^6^A RNA methylation regulators and TB by using “corrr”R packages (version 0.4.5), and the results were visualized and plotted through “ggplot2” and “pheatmap” R packages (version 3.5.2, version 1.0.13respecively). Random forest (RF) algorithm was used to screen and identify TB-related m^6^A RNA methylation regulators Least absolute shrinkage and selection operator (LASSO) regression was performed to construct the prognostic model (19). Receiver Operating Characteristic (ROC) was used to assess the diagnostic performance of the established model.

### 2.3 The correlation between m^6^A RNA methylation regulators and immune features

Single-sample Gene Set Enrichment Analysis (ssGSEA) (20) was employed to assess the relative abundance of specific infiltrating immune cells and the activity of immune responses. This analysis was conducted through the “GSVA”package (version 2.2.0) of R software (version 4.0.2), which employing its built-in ssgsea method for scoring. The list of genes of infiltrated immune cells, including activated CD8+ T cells, natural killer T cells, Regulatory T cells (Tregs), activated dendritic cells, and macrophages were obtained from previous studies (21). The list of immune response genes was obtained from the ImmPort database (22). The correlation between m^6^A RNA methylation regulators and the proportion of immune cells and immune reaction activity was evaluated using Spearman correlation analysis.

### 2.4 Unsupervised clustering analysis of m^6^A modification patterns

Based on the expression profiles of 22 m^6^A RNA methylation regulators, unsupervised consensus clustering analysis was performed on TB samples by using the R package ConsensusClusterPlus (Wilkerson & Hayes, 2010) (23). The clustering analysis employed Euclidean distance as the similarity measure, combined with the k-means algorithm for clustering, and generated a consensus matrix through 1,000 resampling iterations with approximately 80% of samples participating in each iteration to evaluate clustering stability. By comparing the cumulative distribution function (CDF) curves, delta area curves, and consensus heatmaps for different cluster numbers (k = 2–9), the optimal number of clusters was determined. Subsequently, principal component analysis (PCA) was performed based on the expression matrix of the 22 m6A regulators for dimensionality reduction, aiming to visualize the distribution differences between the two modification patterns and validate the clustering effect. Among different m6A modification patterns, the expression levels of m6A regulators, immune cell infiltration abundance, immune response scores, and HLA gene expression were compared. For normally distributed variables, student’s t-test was used for comparisons between the two groups. For non-normally distributed variables, the Mann–Whitney U test (Wilcoxon rank-sum test) was applied. All statistical tests were two-sided, with the significance level set at P < 0.05.

### 2.5 Identification of differentially expressed genes among different modification patterns

To analyze the effect of m^6^A modification patterns on TB, we used the “limma”R packages (version 3.64.1) to analyze the DEGs of the two m^6^A modification patterns. The significance criteria for the determination of DEGs were as |log2FC (fold change) | >0.5 and P.adj< 0.01. Meanwhile, logFC>0.5 and P.ajj <0.01 was defined as upregulated genes. LogFC <-0.5 and P.adj<0.01 was considered as downregulated genes.

### 2.6 Functional enrichment analysis of DEGs

To explore the potential biological functions and signaling pathways of differentially expressed genes (DEGs) under different m^6^A modification patterns, Gene Ontology (GO) and Kyoto Encyclopedia of Genes and Genomes (KEGG) enrichment analysis were performed using the R package clusterProfiler (version 4.16.0). The GO analysis included three categories: Biological Process (BP), Molecular Function (MF), and Cellular Component (CC). For the enrichment analysis, the org.Hs.eg.db database was used as the human gene annotation reference, and all genes detected in the combined GEO dataset were set as the background gene set. Enrichment calculations were conducted using the enrich GO and enrich KEGG functions. Multiple testing correction was applied using the Benjamini–Hochberg method with a significance threshold at adjusted p-value (Padj) < 0.05. Significantly enriched functional categories and signaling pathways were visualized using dot plots (dotplot) and bar plots (barplot).

### 2.7 Gene set enrichment analysis

The “c2.cp.kegg.v7.5.1.entrez.gmt” (25) and “h.all.v 7.5.1. entrez.gmt” (26) data were acquired from Molecular Signatures Database (MLgDB), which was analyzed through GSEA by using R “clusterProfiler”package (24).

### 2.8 Gene set variation analysis and functional annotation

To investigate the biological functional differences between different m^6^A modification-based TB subtypes, Gene Set Variation Analysis (GSVA) was applied to quantitatively evaluate pathway activation levels. The gene sets used in the analysis were collected from the HALLMARK gene sets in the Molecular Signatures Database (MSigDB, https://www.gsea-msigdb.org/gsea/index.jsp) (25). GSVA analysis was performed using the R package “GSVA” (version 2.2.0) (20) and employing a non-parametric kernel method to calculate an enrichment score for each sample in each specific pathway. The main parameters were set as follows: method = “gsva”, kcdf = “Gaussian”, mx.diff = TRUE. Subsequently, the R package “limma” (version 3.64.1) was used to compare the GSVA pathway scores between different m^6^A modification-based TB subtypes. The activation score for each pathway was input as the dependent variable into a linear model for different test without other covariates. To control the false discovery rate (FDR) from multiple hypothesis test, the results were adjusted using the Benjamini–Hochberg method with a corrected FDR < 0.05 as the threshold for statistical significance. The significant differences were visualized with a volcano plot.

### 2.9 Clinical specimens

This case-control investigation included 34 patients with pulmonary tuberculosis who were diagnosed according to clinical laboratory tests including blood, sputum or bronchoalveolar lavage fluid, simple skin tests, and histological findings. 34 age- and sex-matched healthy individuals who underwent a physical examination at the First Affiliated Hospital of Chengdu Medical College were free of diabetes, hypertension, heart, liver, kidney, and other organ diseases or dysfunctions, and had a history of malignant tumors and severe organ dysfunction. None of all TB patients with TB received the standard antituberculosis treatment.

Peripheral venous blood samples were collected from all the participants in the early morning under fasting conditions. Two milliliters of peripheral blood was collected into EDTA anticoagulant tubes. The samples were either immediately analyzed or aliquoted for a single use and stored at -80°C until further use. The study was approved by the Ethics Committee of the First Affiliated Hospital of Chengdu Medical College (Sichuan, China) and conducted in accordance with the Declaration of Helsinki. Written informed consent was obtained from all participants.

### 2.10 Quantitative real-time polymerase chain reaction (QRT-PCR) analysis

Total RNA was isolated from PBMC of TB patients and healthy controls using a whole blood total RNA extraction kit (Simgen, China) according to the manufacturer’s protocols. The concentration and purity of each total RNA were detected (using the A260/A280 and A260/A230 ratios) through a NaoDrop ND-1000 spectrophotometer (Invitrogen). For PCR analysis, 1 mg of total RNA was used to synthesize cDNA by reverse transcription using a PrimeScript TM RT reagent kit (Tiangen Biotech, China) following the manufacturer’s protocol. The product was used as a template for PCR in a CFX-96 real-time PCR system that employed SYBR VRPremix Ex TaqTM II (Tiangen Biotech, China). The primer sequences used for amplification are listed in **Table 2**. Relative expression of each gene was determined using the 2^-△△Ct^ method.

**Table 2:** The amplification primers sequences of different genes.

Gene	Sequence (5’-3’)
METTL3	F:TTGTCTCCAACCTTCCGTAGT
	R:CCAGATCAGAGAGGTGGTGTAG
RBM15	F:ACGACCCGCAACAATGAAG
	R:GGAAGTCGAGTCCTCACCAC
RBM15B	F:TACACGGAGGCTACCAGTACA
	R:GTCGTACAGCCCGTAGTAGTC
CBLL1	F:TCCTTGGGTGGTCTTGATGTT
	R:CAGGTTTCGCTTTGTTTGCTT
YTHDC1	F:AACTGGTTTCTAAGCCACTGAGC
	R:GGAGGCACTACTTGATAGACGA
YTHDC2	F:AGGACATTCGCATTGATGAGG
	R:CTCTGGTCCCCGTATCGGA
HNRNPC	F:TCCTCCTCCTATTGCTCGGG
	R:GTGTTTCCTGATACACGCTGA
HNRNPA2B1	F:ATTGATGGGAGAGTAGTTGAGCC
	R:AATTCCGCCAACAAACAGCTT
IGF2BP1	F:GCGGCCAGTTCTTGGTCAA
	R:TTGGGCACCGAATGTTCAATC
IGF2BP3	F:ACGAAATATCCCGCCTCATTTAC
	R:GCAGTTTCCGAGTCAGTGTTCA
ELAVL1	F:GGGTGACATCGGGAGAACG
	R:CTGAACAGGCTTCGTAACTCAT
LRPPRC	F:GCTCATAGGATATGGGACACACT
	R:CCAGGAAATCAGTTGGTGAGAAT
LAMP2	F:GAAAATGCCACTTGCCTTTATGC
	R:AGGAAAAGCCAGGTCCGAAC

### 2.11 Statistical analysis

All data analyses were performed in R software (version 4.0.2). For gene expression analysis, after normalization and log transformation, the data approximated a normal distribution under the large sample assumption. Therefore, differential analysis was conducted using the limma package (version 3.64.1). For comparisons of continuous variables between independent samples, student’s t-test was applied when the data met the assumptions of normal distribution and homogeneity of variance. If the variables did not follow a normal distribution (e.g., Shapiro–Wilk test p < 0.05) or the sample size was small with markedly skewed distribution, the Mann–Whitney U test was used. All statistical tests were two-sided, and a p-value < 0.05 was considered statistically significant.

The expression correlations among m6A regulators were calculated using Spearman’s rank correlation analysis. The analysis was based on the paired expression values of each regulator, and the results were expressed as correlation coefficients (ρ). The significance of correlations was determined based on p-values without adjustment for multiple tests. The correlation coefficient matrix and the significance results were visualized via heatmaps and network plots.

The receiver operating characteristic (ROC) curve and area under the ROC curve (AUC) were calculated to evaluate the feasibility of m^6^A regulators as potential markers for TB diagnosis. Meanwhile, the discriminative performance of the model was evaluated using the ROC curve. To compare the predictive ability of the model across different datasets, ROC curves were calculated based on the training set and the validation set, respectively. The area under the curve (AUC) and its 95% confidence interval were computed using the R package “pROC” (Version 1.18.5). To assess the robustness of the model, the study samples were randomly divided into a training set and a validation set at a ratio of 7:3 for model fitting and validation without additional resampling or cross-validation.

## 3 Results

### 3.1 The differential expression of m^6^A -related genes in TB

**Figure 2:**
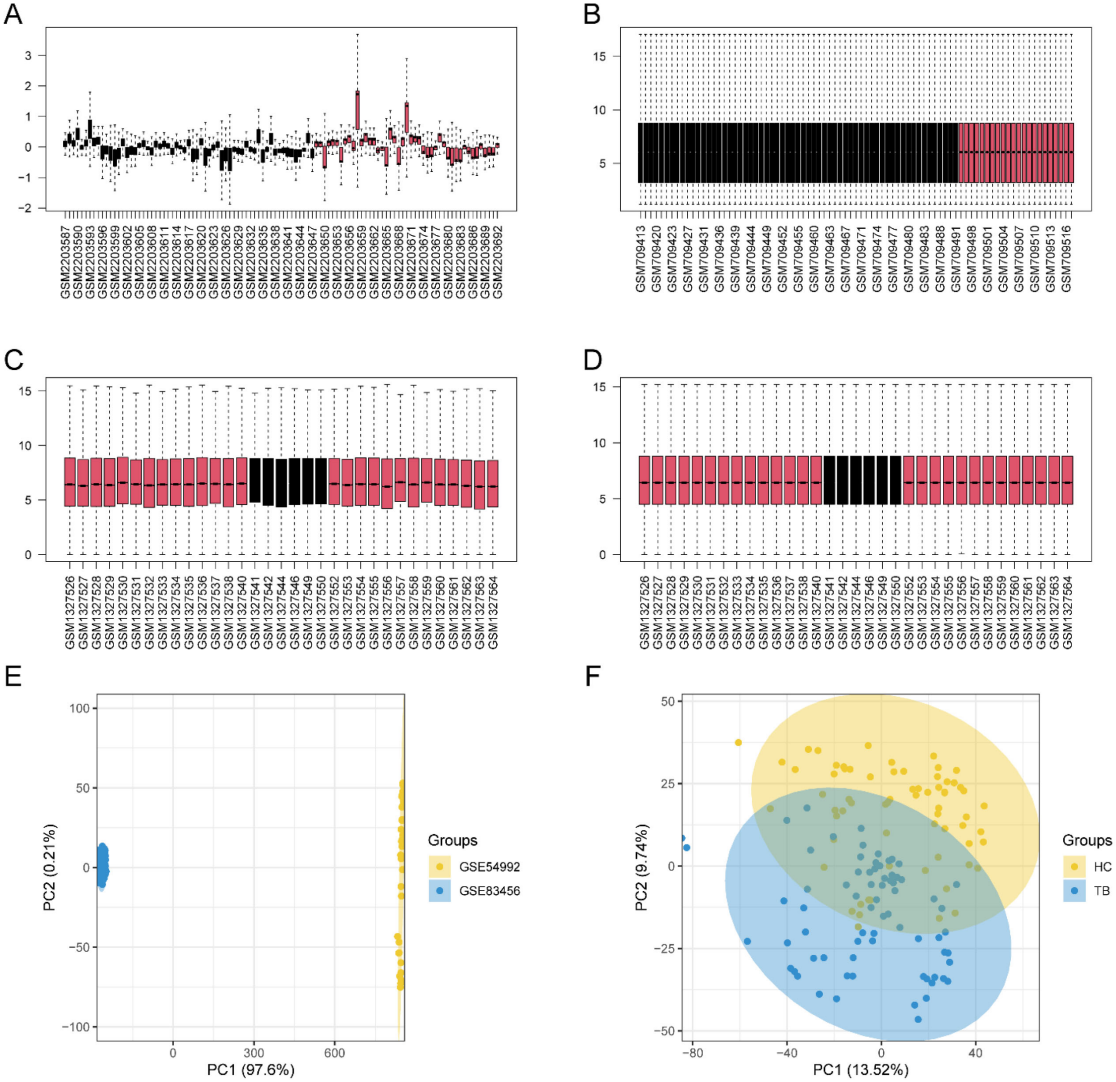
Normalized expression matrices **(A-D)** and PCA diagrams **(E, F)** of the GSE54992, GSE83456 datasets. PCA, principal component analysis.

The workflow of this study is illustrated in **Figure 1**. After the integration of the GEO datasets GSE54992 and GSE83456, the raw expression data were first subjected to background correction and normalization using “limma”R packages (version 3.64.1), followed by batch-effect adjustment via the empirical Bayesian algorithm implemented in the “sva” package to ensure comparability across datasets. The data of boxplots of normalized expression values demonstrated that after normalization, the median levels of the boxplots were well aligned, indicating comparable distributions across samples (**Figures 2A-D**). Subsequently, principal component analysis (PCA) was conducted to evaluate overall clustering characteristics and group consistency. The PCA results (**Figures 2E, F**) revealed clear separation between TB group and healthy control group in the principal component space. PC1 and PC2 explained 13.52% and 9.74% of the total variance, respectively, indicating that the first two principal components can effectively capture the primary variation structure among the samples.

**Figure 3:**
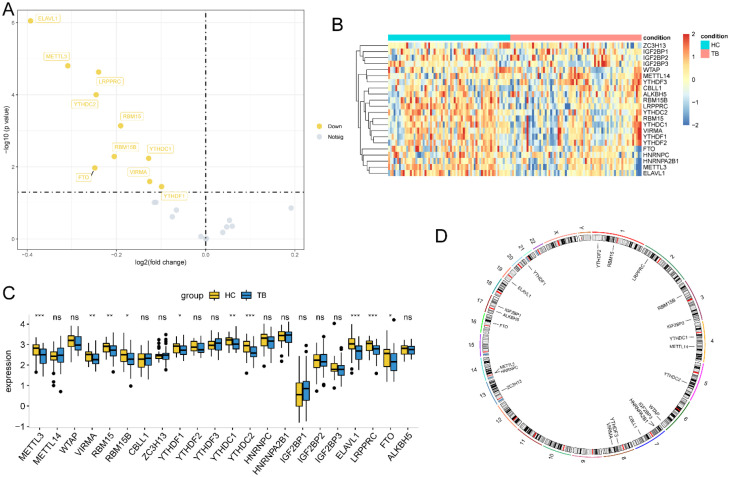
The differential expression of m^6^A RNA methylation genes between healthy controls and TB cases. **(A-D)** Volcano plot, Heat map, box plot and chromosome map of 22 m^6^A RNA methylation regulators between healthy control samples and TB samples, respectively. (*P<0.05; ***P<0.001; ****P<0.0001. ns, p>0.05).

Then, the landscape of genetic expression of m^6^A RNA methylation genes between the TB group and control group was analyzed through “limma” R packages. Volcano plots of DEGs in the above two datasets showed that METTL3, VIRMA, RBM15, RBM15B, YTHDF1, YTHDC1, YTHDC2, ELAVL1, LRPPRC and ALKBH5 were downregulated in TB (**Figure 3A**). The data further demonstrated that the results of the heat map (**Figure 3B**), box plot (**Figure 3C**) and chromosome map (**Figure 3D**) were the same as those of the volcano plot.

**Figure 4:**
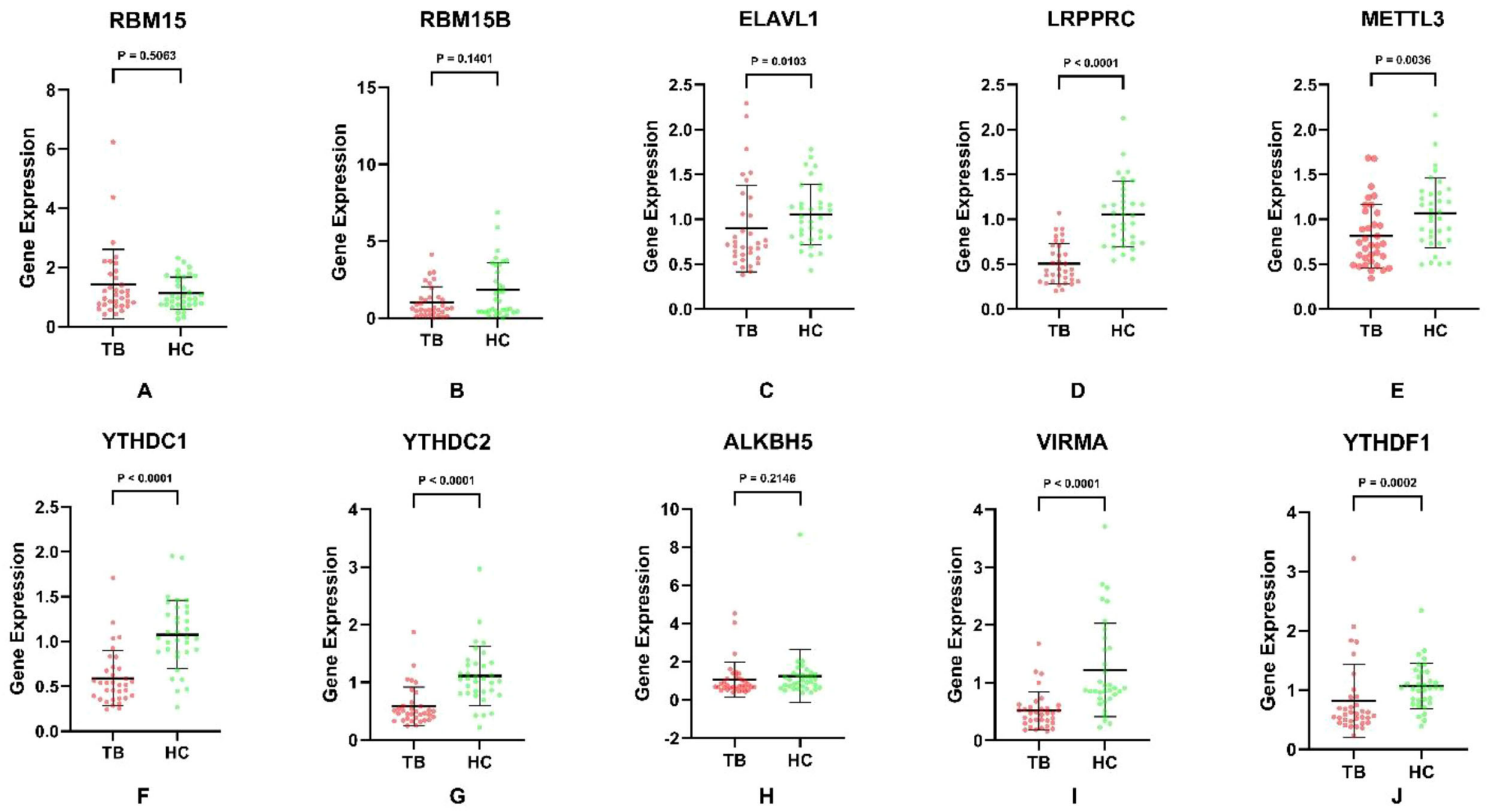
The mRNA expression of METTL3, VIRMA, RBM15, RBM15B, YTHDF1, YTHDC1, YTHDC2, ELAVL1, LRPPRC and ALKBH5 in peripheral blood by using qRT-PCR in our collected samples. **(A–J)** The expression of RBM15,RBM15B,ELAVL1,LRPPRC,METTL3,YTHDC1,YTHDC2,ALKBH5,VIRMA and YTHDF1 in TB samples and healthy control samples,respectively.

**Figure 5:**
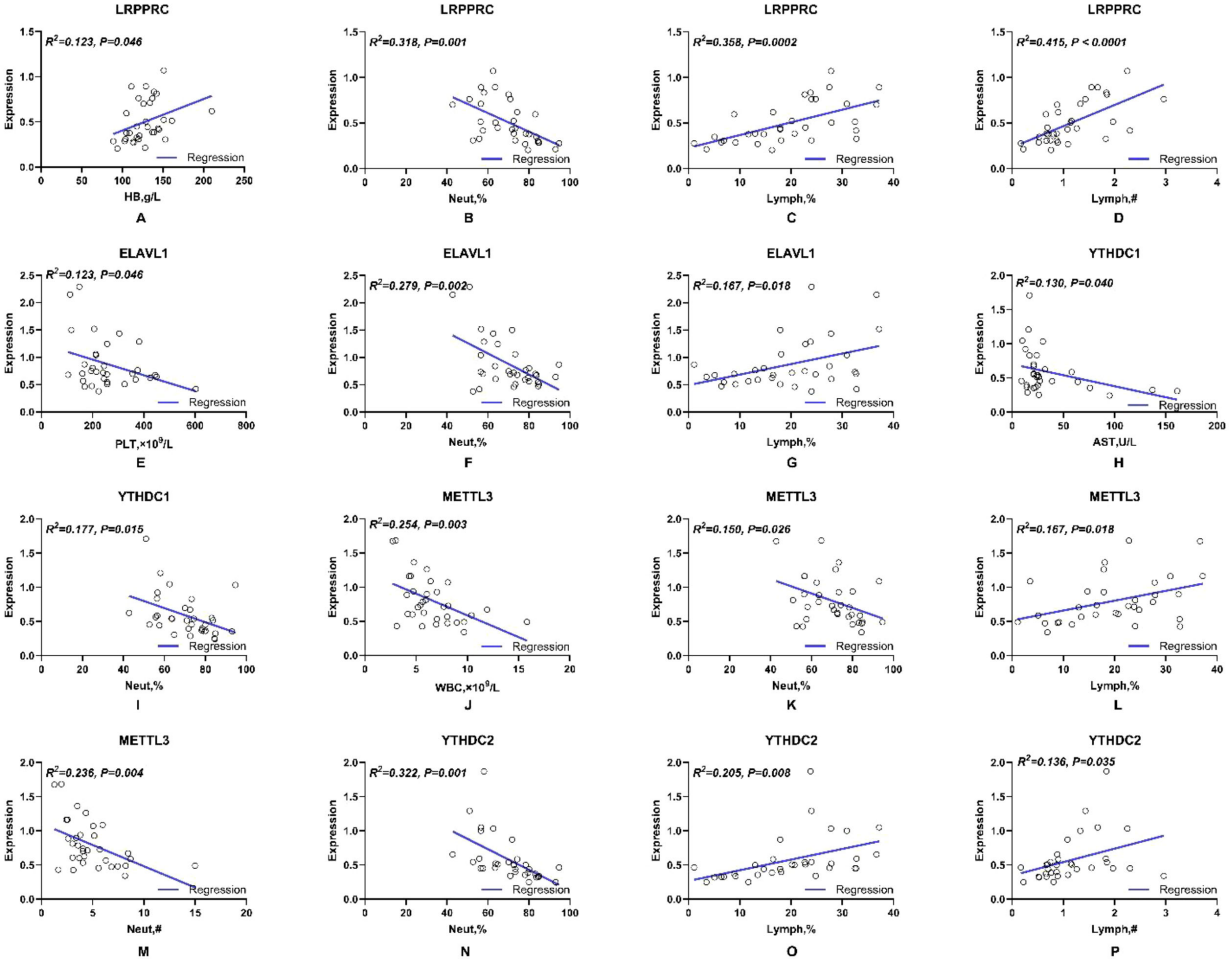
The spearman correlation analysis between m^6^A genes and laboratory indicators in our collected TB specimens. **(A-P)** Statistically significant correlation analysis of serum *LRPPRC, ELAVL1, YTHDC1, METTL3, YTHDC2* with different laboratory makers respectively in TB patients.

Moreover, the validation and clinical relevance of different m^6^A regulators in TB were evaluated. We examined the mRNA expression of METTL3, VIRMA, RBM15, RBM15B, YTHDF1, YTHDC1, YTHDC2, ELAVL1, LRPPRC and ALKBH5 in peripheral blood by using qRT-PCR. METTL3, VIRMA, YTHDF1, YTHDC1, YTHDC2, ELAVL1 and LRPPRC mRNA levels were significantly downregulated in TB samples compared to those in the control group (**Figures 4A-J**). In addition, we evaluated the association between serum METTL3, VIRMA, YTHDF1, YTHDC1, YTHDC2, ELAVL1, LRPPRC mRNA expression, and clinical markers in TB patients. As shown in **Figure 5**, there was a close association between LRPPRC and Lymphocyte percentage (Lymph%) (r=0.358, P = 0.0002) and number (Lymph#) (r=0.415, P<0.0001) (**Figures 5A-P**).

### 3.2 The expression correlation analysis of m^6^A regulators in TB

**Figure 6:**
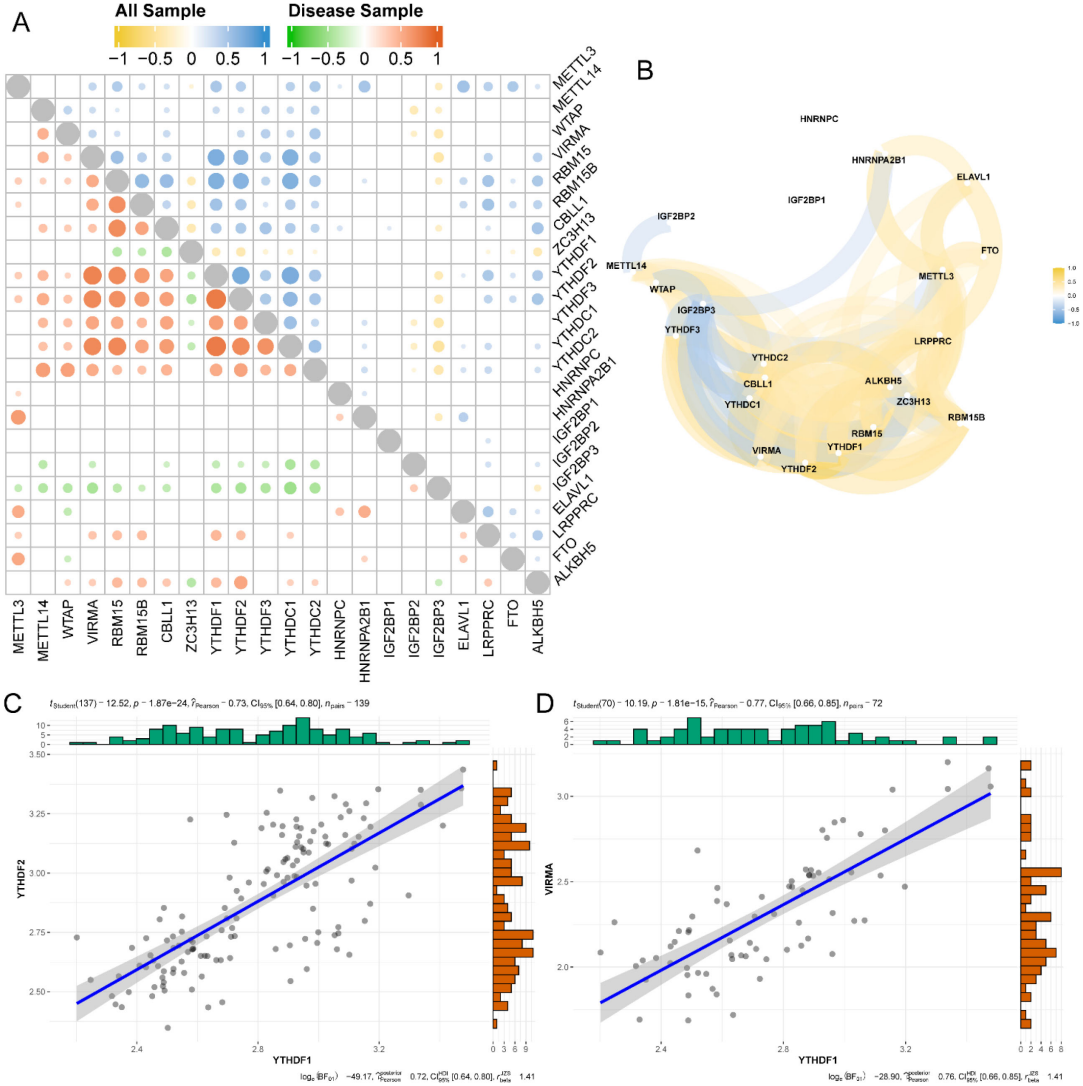
The correlation analysis of m^6^A regulators in TB. **(A)** The expression correlation heat map of 22 m^6^A gene. The right upper corner is the all samples, and the left lower corner is TB cases. **(B)** The network between m^6^A regulators. **(C)** The scatter plot of YTHDF2 and YTHDF1 expression in all samples. **(D)** The scatter plot of YTHDF1 and VIRMA expression in TB samples.

The correlation between the expression levels of the 22 m^6^A genes in TB and normal samples was analyzed. The results were visualized using a heat map (**Figure 6A**) and network map (**Figure 6B**). The upper right section presents the expression correlation of m^6^A -related genes in all samples, whereas the lower left section shows the expression correlation of m^6^A -related genes in TB samples. These data revealed that YTHDF2 was strongly associated with YTHDF1 in the TB group (**Figure 6C**). YTHDF1 was highly correlated with VIRMA in all the samples ((**Figure 6D**).

### 3.3 The prediction model of m^6^A regulators in TB

**Figure 7:**
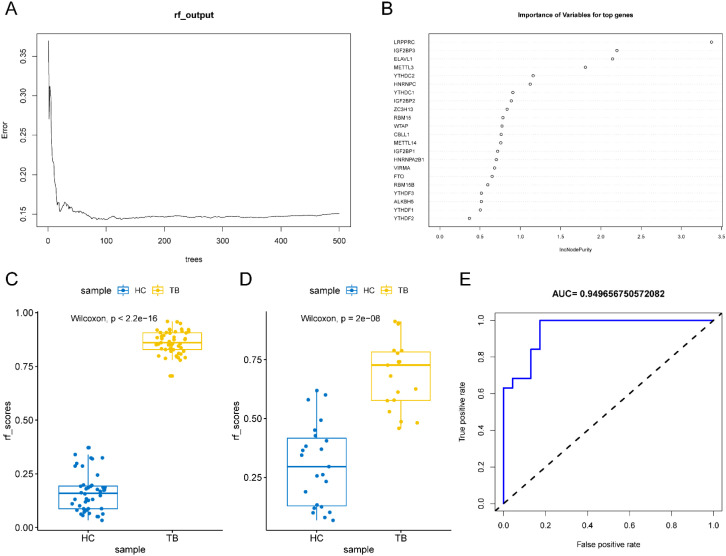
Random forest analysis. **(A, B)** Modeling of m^6^A gene through random forest method in TB. **(C, D)** The box plot of training group and verification group. **(E)** ROC curve of random forest.

To further explore the diagnostic ability of m^6^A -related regulators in TB, the random forest method was used (**Figures 7A, B**). The samples were randomly divided into the training (70%) and validation (30%) sets. Boxplots (**Figures 7C, D**) revealed significant differences in the model scores between the TB and healthy groups in both the training and validation sets. The ROC curve (**Figure 7E**) demonstrated that the constructed model exhibited excellent diagnostic performance for TB, indicating that m^6^A genes had strong predictive power.

**Figure 8:**
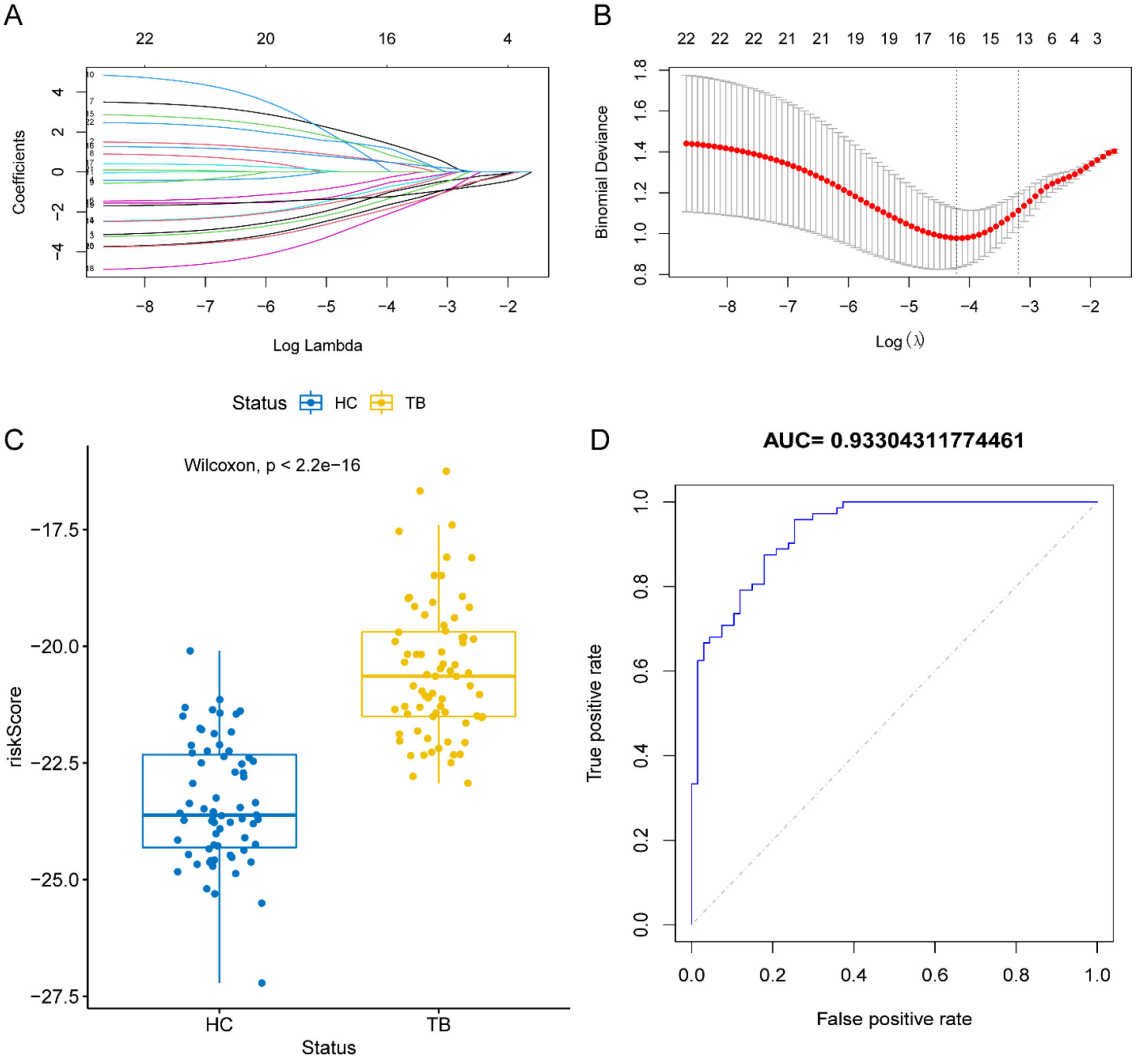
LASSO regression modeling. **(A, B)** LASSO regression was used to model of m^6^A gene. **(C)** Score box plot. **(D)** Diagnostic ROC curve of LASSO regression.

LASSO regression analysis was used to screen variables and construct a prediction model as follows: risk scores = METTL3 × (-1.265) + METTL14 × 0.598 + WTAP × (-1.559) + RBM15 × (-0.926) + RBM15B × (-0.432) + CBLL1 × 1.623 + YTHDF2 × 0.564 + YTHDC1 × (-0.744) + YTHDC2 × (-1.844) + HNRNPC × (-1.119) + HNRNPA2B1 × 1.109 + IGF2BP1 × 0.559 + IGF2BP3 × (-2.372) + ELAVL1 × (-1.273) + LRPPRC × (-2.031) + ALKBH5 × 1.297. As showed in **Figures 8A, B**, the results were the same as those in the above conclusion. The boxplot shows a significant difference in the risk scores between the TB and healthy groups (**Figure 8C**). The ROC curve (**Figure 8D**) indicated that the risk model had strong diagnostic capability for TB.

**Figure 9:**
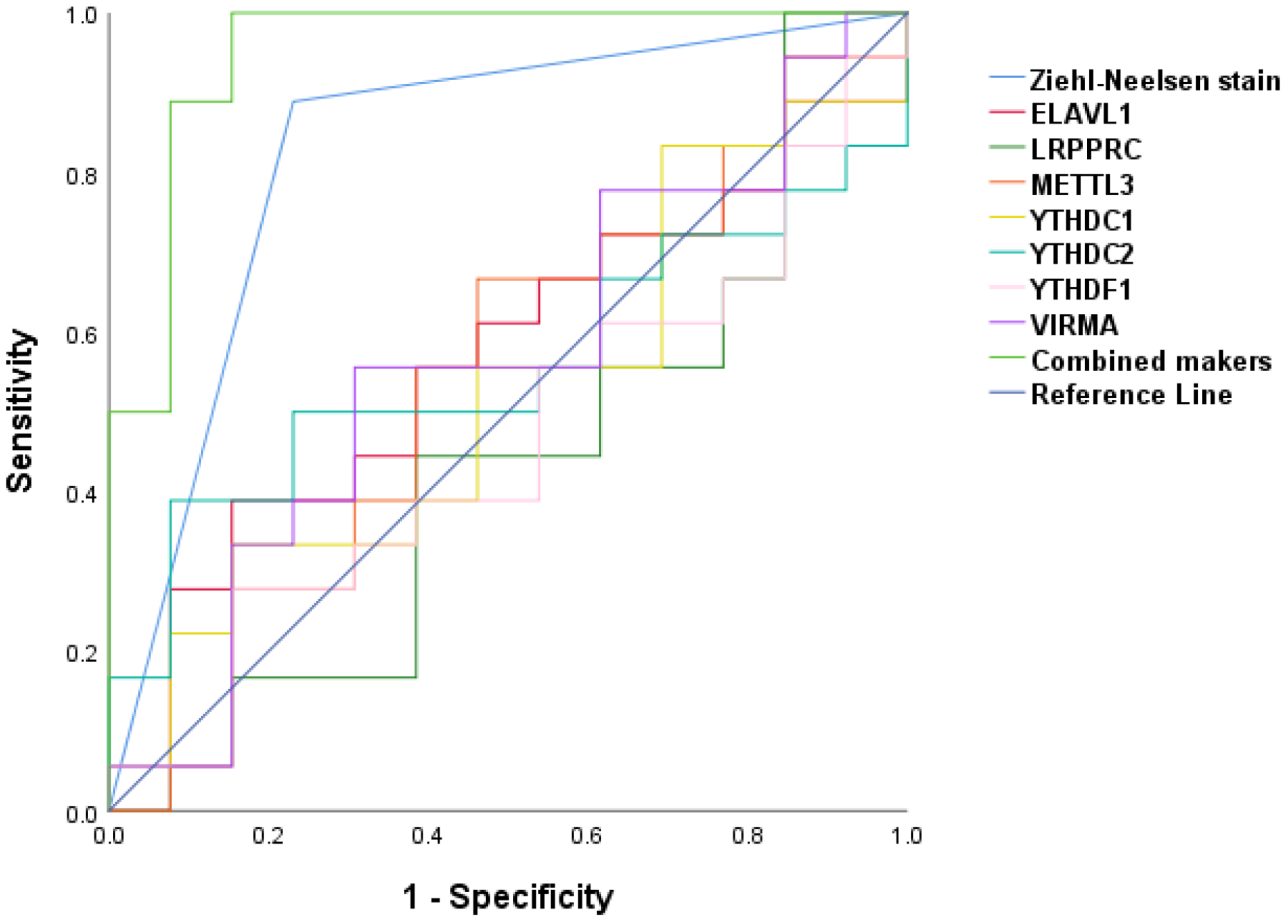
ROC revealing a diagnostic value of METTL3, VIRMA, YTHDF1, YTHDC1, YTHDC2, ELAVL1and LRPPRC combination with Ziehl-Neelsen staining as TB infection biomarker.

In addition, the predictive values of METTL3, VIRMA, YTHDF1, YTHDC1, YTHDC2, ELAVL1and LRPPRC individually and in combination with Ziehl-Neelsen staining were evaluated by using a binary logistic regression model. Receiver operating characteristic (ROC) curves were plotted to analyze and compare their predictive values (**Figure 9**). The areas under the ROC for predicting TB using METTL3, VIRMA, YTHDF1, YTHDC1, YTHDC2, ELAVL1and LRPPRC were 0.543 (95% CI: 0.331–0.755), 0.564 (95% CI: 0.354–0.774), 0.462 (95% CI: 0.254–0.669), 0.521(CI:0.313-0.730), 0.556(CI:0.350-0.761), 0.564(CI:0.358-0.77) and 0.436(CI:0.222-0.649) respectively, indicating that none of the individually identified genes can possess sufficient diagnostic accuracy for clinical application. When compared to individual predictions, the combined ROC area under the curve for these seven markers and Ziehl-Neelsen staining was 0.953 (95% CI: 0.874–1.00) (P<0.001).

### 3.4 Correlation analysis of m^6^A regulators with immune cell and immune process in TB

**Figure 10:**
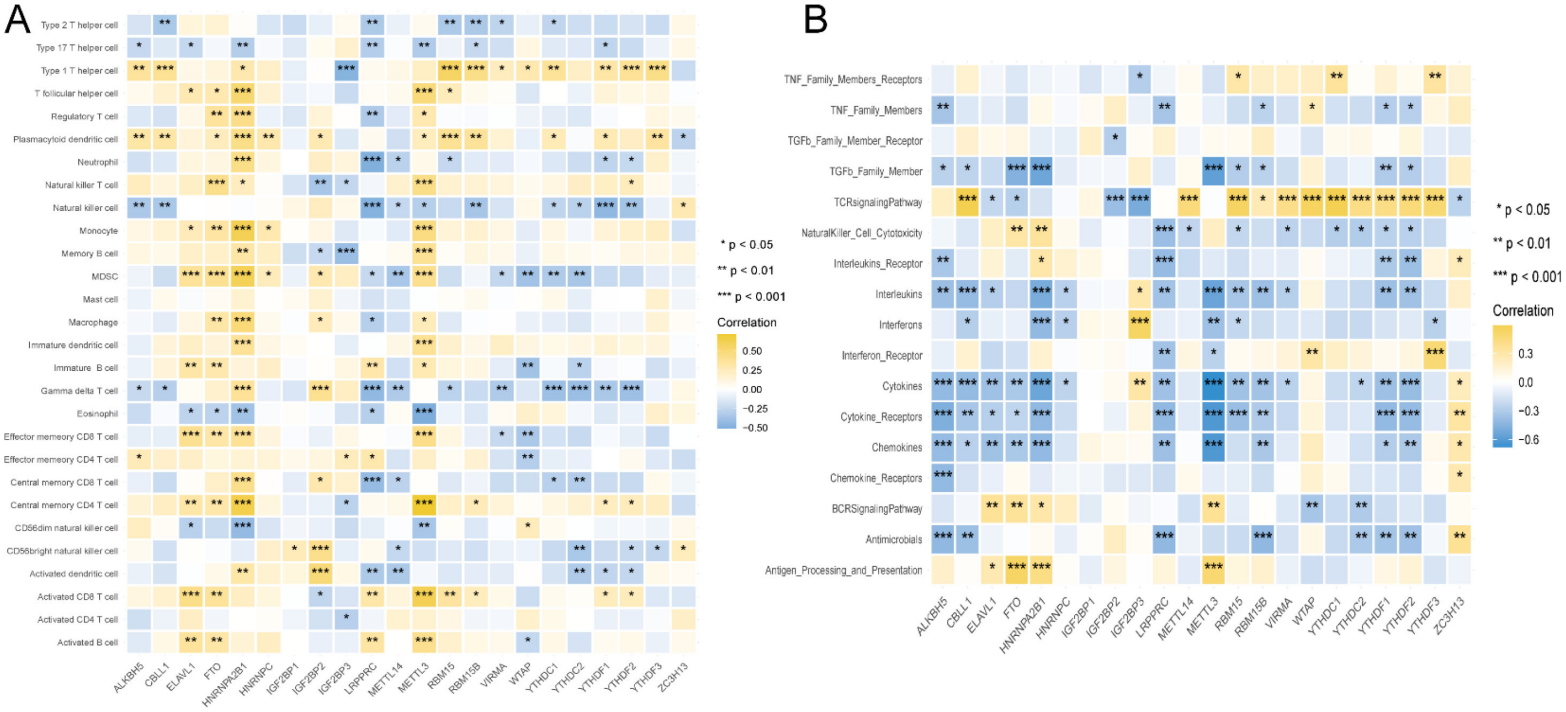
The Correlation between infiltrating immune cells, immune response genes and m^6^A regulators. **(A)** The association between abnormal infiltrating cell in the immune microenvironment and abnormal m^6^A regulators displayed by dot plot. **(B)** The dot plot showed the correlation between each immune response pathway and m^6^A regulators.

Immune cell infiltration is a vital component of the tumor microenvironment and is closely associated with the development of various diseases (Gajewski et al., 2013). Therefore, the relationship between m^6^A regulators, immune cell infiltration, and immune response was explored in TB (**Figures 10A, B**). FTO and METTL3 were positively correlated with most immune cells, whereas LRPPRC exhibited a negative correlation. Type 1 T helper cells were positively associated with most m^6^A genes, whereas gamma delta T cells were negatively correlated. In terms of immune processes, ALKBH5 and METTL3 levels were negatively correlated with immune processes. These findings suggested that m^6^A regulators could serve as predictors of the immune microenvironment and immune processes in TB, with METTL3 acting as a significant immunosuppressive regulator, and HNRNPA2B1 acting as an immune-activating gene.

### 3.5 m^6^A regulators-mediated m^6^A modification patterns in TB

**Figure 11:**
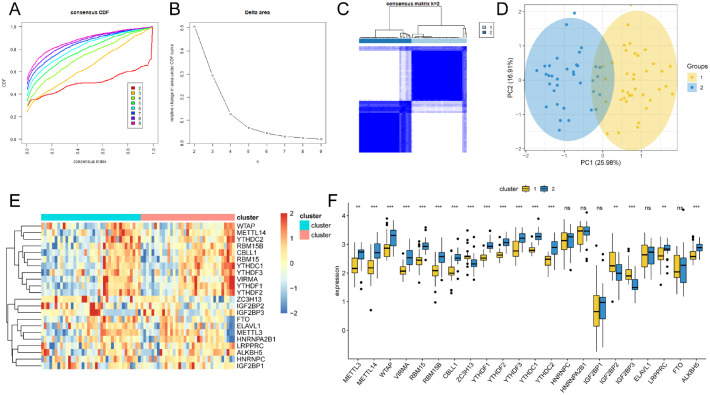
The unsupervised cluster analysis of 22 m^6^A regulators. Two different subtypes of m^6^A modification patterns were identified in TB. **(A)** The distribution cumulative of consensus clustering when k=2-9. **(B)** Relative change of area under CDF curve when k=2-9. **(C)** Heat map of the co-occurrence proportion matrix of TB samples when k=2. **(D)** The transcriptome difference in two modification modes. **(E)** The heat map of 22 m^6^A regulators in two modification modes. **(F)** The expression of 22 m^6^A regulators in two m^6^A subtypes. ns:p>0.05, *:p<0.05, **:p<0.01, ***:p<0.001.

Based on the expression of 22 m^6^A regulators, unsupervised cluster analysis was applied to classify the different m^6^A modification patterns in TB (**Figures 11A-C**). The data demonstrated that the optimal clustering stability was k = 2 in the consensus clustering. Patients with TB were divided into two subtypes (clusters 1 and 2). Furthermore, PCA revealed a statistically significant difference between the two m^6^A molecular subtypes (**Figure 11D**). Heat maps ((**Figure 11E**) and boxplots ((**Figure 11F**) demonstrated the expression specificity of m^6^A regulators between these two molecular subtypes.

### 3.6 Immune microenvironment characteristics of different m^6^A modification patterns

**Figure 12:**
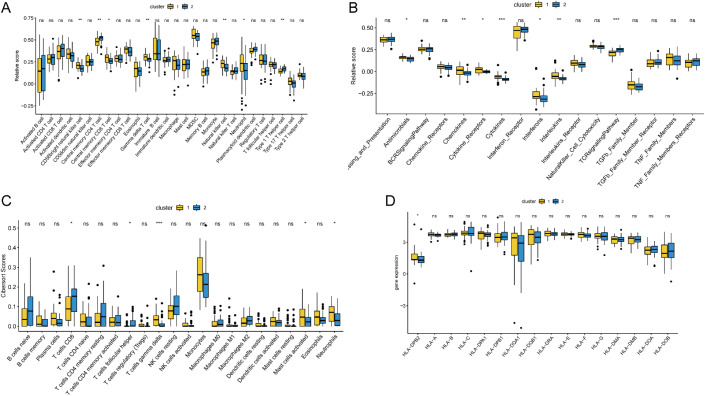
Differences in immune microenvironment characteristics between different m^6^A modification patterns. **(A)** The differences abundance of infiltrating immune cells in different immune microenvironments by ssgesa scores under two m^6^A modification patterns. **(B)** The differences of immune processes scores under two m^6^A modification patterns. **(C)** The abundance difference of infiltrating immune cells in different immune microenvironments by CIBERSORT scores under two m^6^A modification patterns. **(D)** Different expression of HLA genes in three m^6^A modification patterns. ns:p>0.05, *:p<0.05, **:p<0.01, ***:p<0.001.

m^6^A modification may affect the translation efficiency or stability of immune-related genes, leading to differences in immune response intensity between the two subtypes. To explore the differences in immune microenvironment features between different m^6^A modification patterns, we evaluated immune cell infiltration, immune response, and HLA expression under different m^6^A modification patterns. As shown in **Figure 12A**, the number of immune cells differed between the two groups. Pattern 1 exhibited a relatively high proportion of activated immune cells, including gamma delta T cells, neutrophils, and NK cells. Pattern 2 showed a higher proportion of type 1 T-helper cells. Chemokine and cytokine processes were relatively more active in pattern 1, whereas the TCR signaling pathway was highly active in pattern 2 (**Figure 12B**), which aligns with previous analysis of immune processes. To enhance the reliability of the results, CIBERSORT was used to calculate the immune cell content in the different modes (**Figure 12C**). Similar to ssGSEA, there was a high level of Gamma Delta T cells in Pattern 1. However, there was no significant difference in HLA family gene expression between the two patterns (**Figure 12D**). These findings suggested that pattern 1 mediates an active immune response, whereas pattern 2 regulates a mild immune response, revealing the important role of m^6^A methylation in regulating the formation of different immune microenvironments in TB.

### 3.7 Biological features of different m^6^A modification patterns

**Figure 13:**
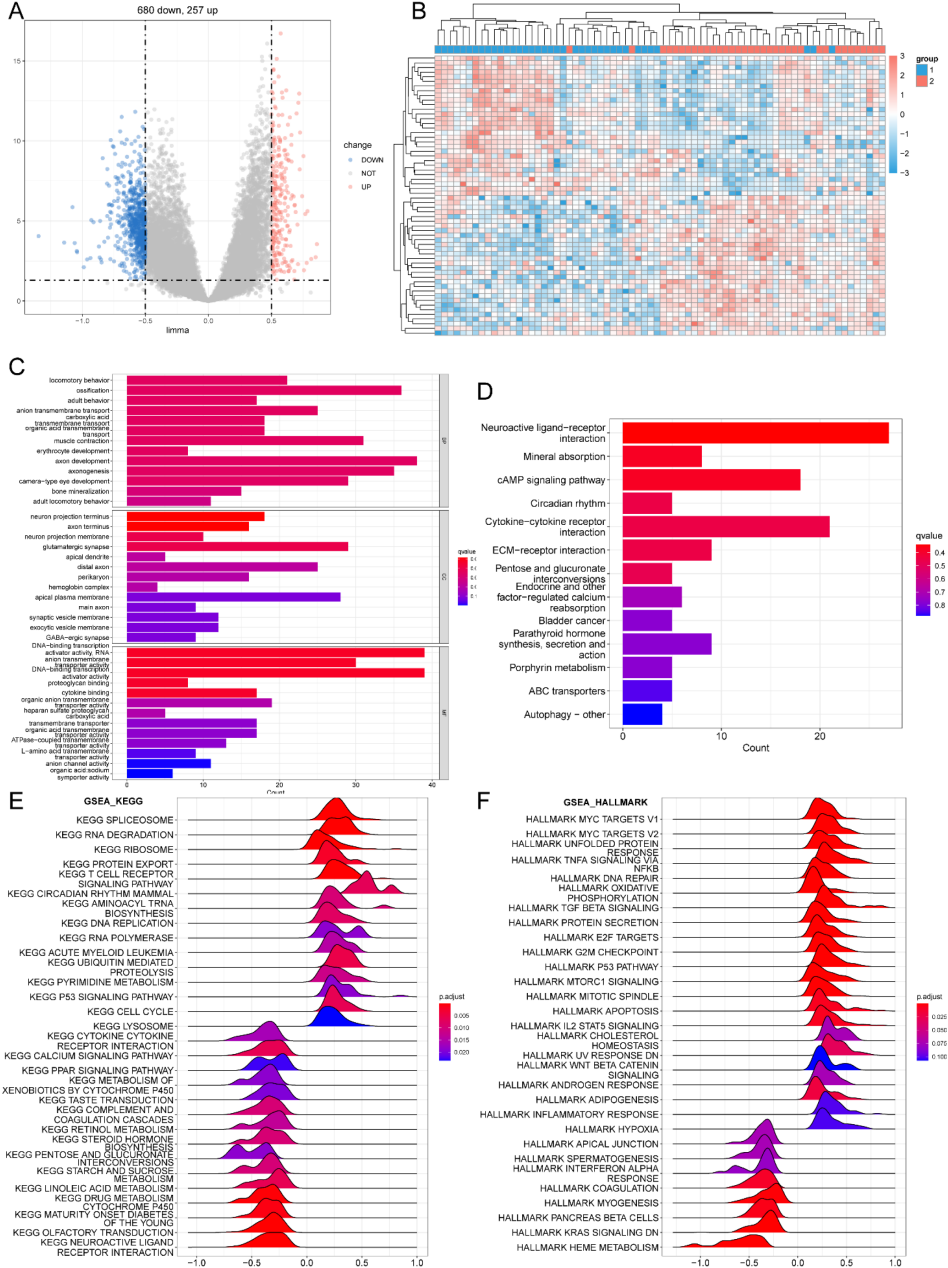
Difference analysis and enrichment analysis of two m^6^A modification patterns. **(A)** Volcano map of m^6^A modification Model 1 and Model 2. **(B)** Heat map of m^6^A modification mode 1 and Mode 2. **(C)** GO enrichment analysis between pattern 1 and pattern 2. **(D)** KEGG enrichment analysis of m^6^A modified pattern 1 and pattern 2. **(E, F)** GSEA enrichment analysis of m^6^A modified pattern 1 and pattern 2.

The two subtypes may represent different biological states. We use “limma” package to investigate the biological responses under the two m^6^A modification patterns. Volcano and heat maps were used to display the results of the difference analysis (**Figures 13A, B**). We set the thresholds for differentially expressed genes (DEGs) as |log2 fold change (logFC)| > 0.5 and adjusted P-value (Padj) < 0.01. Enrichment analysis was conducted on the DEGs (**Figures 13C, D**). GSEA was used to conduct an enrichment analysis for the two patterns (**Figures 13E, F**). The KEGG and HALLMARK results showed that pattern 2 was more biologically active (**Tables 3**, **4**).

**Table 3:** GO function enrichment analyses on the difference genes between m^6^A subtypes.

Ontology	ID	Description	p.adjust
BP	GO:0007626	locomotory behavior	0.051255
BP	GO:0001503	ossification	0.051255
BP	GO:0030534	adult behavior	0.051255
BP	GO:0098656	anion transmembrane transport	0.051255
BP	GO:1905039	carboxylic acid transmembrane transport	0.051255
BP	GO:1903825	organic acid transmembrane transport	0.051255
BP	GO:0006936	muscle contraction	0.051255
BP	GO:0048821	erythrocyte development	0.051255
BP	GO:0061564	axon development	0.05224
BP	GO:0007409	axonogenesis	0.05224
BP	GO:0043010	camera-type eye development	0.052321
CC	GO:0044306	neuron projection terminus	0.017179
CC	GO:0043679	axon terminus	0.017179
CC	GO:0032589	neuron projection membrane	0.041449
CC	GO:0098978	glutamatergic synapse	0.041449
MF	GO:0001228	DNA-binding transcription activator activity, RNA polymerase II-specific	0.026143
MF	GO:0008509	anion transmembrane transporter activity	0.026143
MF	GO:0001216	DNA-binding transcription activator activity	0.026143
MF	GO:0043394	proteoglycan binding	0.028116
MF	GO:0019955	cytokine binding	0.028116

**Table 4:** KEGG function enrichment analyses on the difference genes between m^6^A subtypes.

ID	Description	p.value
hsa04080	Neuroactive ligand-receptor interaction	0.001174
hsa04978	Mineral absorption	0.002827
hsa04024	cAMP signaling pathway	0.003954
hsa04710	Circadian rhythm	0.00777
hsa04060	Cytokine-cytokine receptor interaction	0.009087
hsa04512	ECM-receptor interaction	0.009411
hsa00040	Pentose and glucuronate interconversions	0.011519
hsa04961	Endocrine and other factor-regulated calcium reabsorption	0.020131
hsa05219	Bladder cancer	0.024631
hsa04928	Parathyroid hormone synthesis, secretion and action	0.028719
hsa00860	Porphyrin metabolism	0.029631
hsa02010	ABC transporters	0.035228
hsa04136	Autophagy – other	0.03976

### 3.8 TB subtypes construction based on differentially expressed genes of m^6^A modification patterns

**Figure 14:**
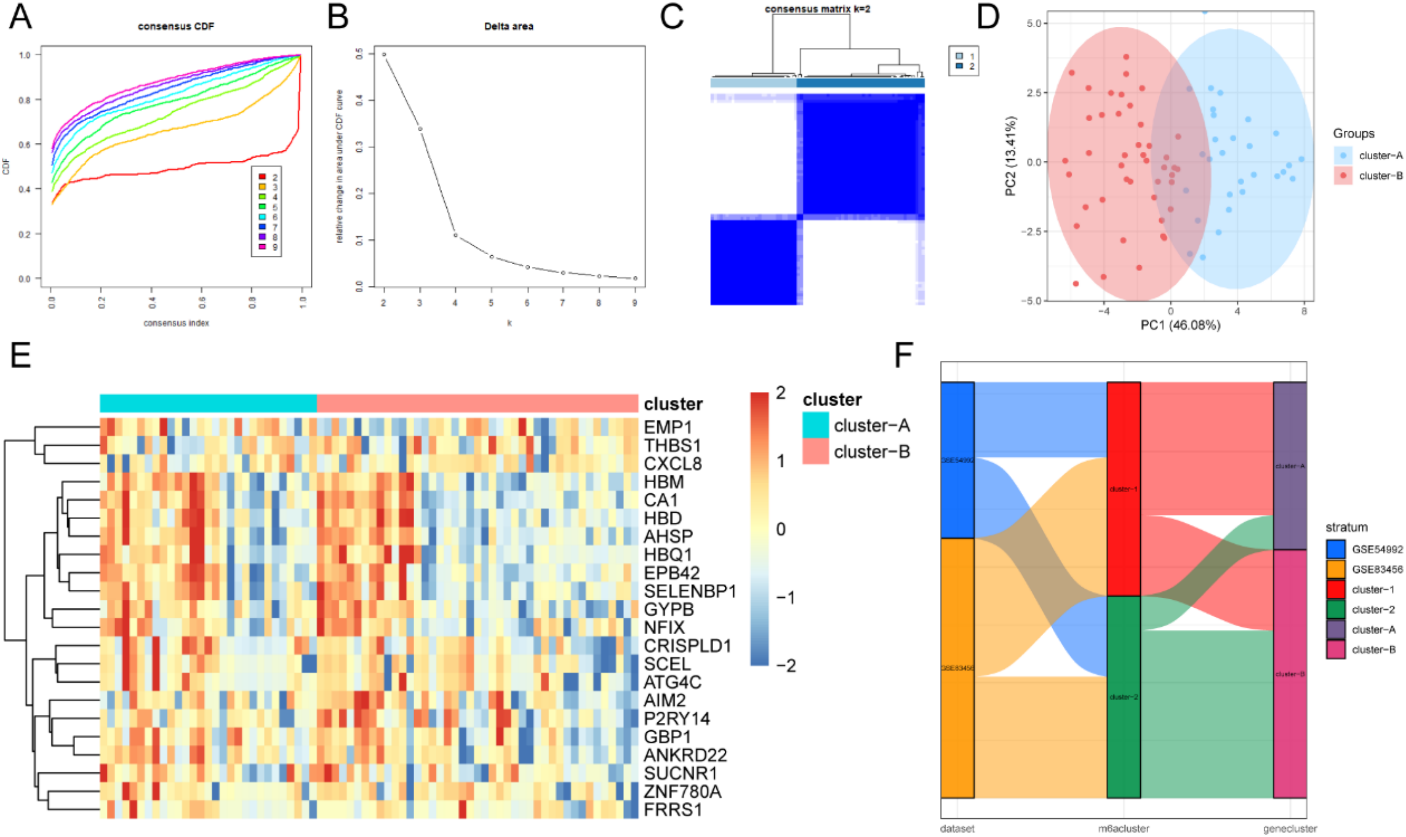
Construction of TB subtypes under different m^6^A modification patterns. **(A)** The cumulative distribution function of consensus clustering when k=2-9. **(B)** Relative change of area under CDF curve when k=2-9. **(C)** Heat map of the co-occurrence proportion matrix of TB samples when k=2. **(D)** The component analysis was performed in transcriptome maps of different TB subtype. **(E)** The expression heat map of 22 m^6^A related characteristic genes in two modification modes. **(F)** Sankey diagram of two typing construction processes.

To further elucidate the impact of m^6^A modification on the transcriptomic landscape of tuberculosis (TB), this study conducted a secondary subtype analysis based on differentially expressed genes (DEGs), which was built upon the previously identified m^6^A modification patterns derived from 22 m^6^A regulators. This analysis aimed to extend from the m^6^A regulatory level to the gene expression level and systematically uncovered the downstream effects of m^6^A modification on TB molecular heterogeneity. The clustering results (**Figures 14A–C**) showed that the m6A-related signature genes robustly distinguished two expression subtypes among TB samples. PCA analysis (**Figure 14D**) further validated clear separation between the two subtypes in transcriptomic space, suggesting that m6A modification may drive distinct downstream transcriptional responses. The heatmap (**Figure 14E**) displayed differential expression patterns of the signature genes across the two subtypes, reflecting potential functional divergences in metabolic regulation, immune responses, and signaling pathways. The Sankey diagram (**Figure 14F**) revealed the relationship between the m^6^A modification patterns and expression subtypes, indicating that different m^6^A modification patterns may shape distinct transcriptional states by regulating specific gene networks.

### 3.9 Functional differences in TB subtypes

**Figure 15:**
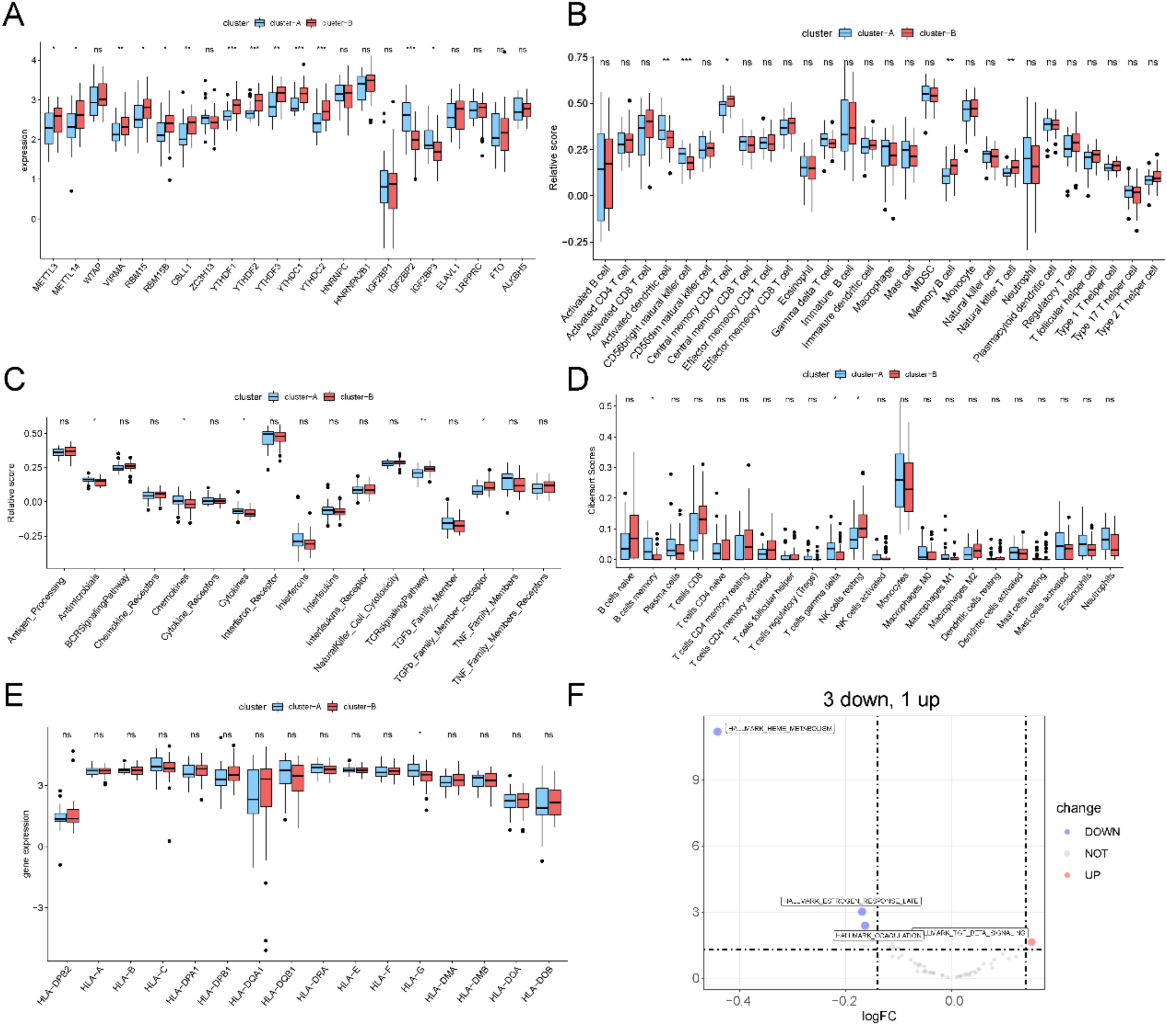
Functional differences of TB subtypes in different m^6^A modification modes. **(A)** The m^6^A gene expression difference of TB subtypes in two m^6^A modification modes. **(B)** Under the TB subtype, the abundance difference of infiltrating immune cells in different immune microenvironments was evaluated by ssgesa scores. **(C)** The score differences of immune process in TB subtype. **(D)** The abundance of infiltrating immune cells in different immune microenvironments was performed by CIBERSORT score under two different TB subtypes. **(E)** different expression of HLA genes in two TB subtypes **(F)** the volcano map of different HALLMARK pathway between two TB subtype. ns:p>0.05, *:p<0.05, **:p<0.01, ***:p<0.001.

As shown in **Figure 15A**, no significant differences were observed in immune cell infiltration, immune processes, or expression of HLA family genes (**Figures 15B-E**), suggesting that the differences between the two subtypes might not be driven by immune infiltration. We then employed the GSVA package to convert the expression matrix into a pathway activation score matrix in the “h.all.v7.5. symbols” gene set. The R package limma was used to compare pathway activation scores between the two subtypes, and a volcano plot was generated (**Figure 15F**). Subtype A primarily activates the hallmark heme metabolism pathway, whereas subtype B mainly activates the hallmark TGF beta signaling pathway.

## 4 Discussion

Numerous reports have revealed that m^6^A regulators are involved in many diseases such as cancers, cardiovascular disorders, abdominal aortic aneurysm and chronic obstructive pulmonary disease. For example, m^6^A can shape the host response to viral infections, such as SARS-CoV-2 (27), pseudorabies virus infection (28) and bacterial infection (29) by modulating the stability and translation of immune-related transcripts, which has attracted extensive attention for exploring m^6^A in TB. However, m^6^A regulators in TB field still remain poorly understood. From my perspective, our conclusions provide scientific investigation and experiments for m^6^A regulators in TB, which will help us find reliable directions for future experimental research on TB and novel targets for effective therapies.

In the present study, we present a comprehensive analysis of the important role of m^6^A modification in TB, combined with clinical validation. First, we identified significant downregulation of multiple crucial m^6^A regulators (METTL3, VIRMA, YTHDF1, YTHDC1, YTHDC2, ELAVL1, and LRPPRC) in the peripheral blood of TB patients, which was consistent between GEO datasets and subsequently confirmed by qRT-PCR, suggesting global dysregulation of m^6^A during TB pathogenesis. Our data, consistent with previous studies, revealed that the expression of YTHDF1, ELAVL1, LRPPRC, and HNRNPC mRNA was obviously downregulated in TB patients compared to healthy control (30, 31). Meanwhile, in PTB, studies have demonstrated that FTO gene polymorphisms are closely connected with PTB, and the expression of ALKBH5 and FTO levels are reduced in PTB patients (32). Another report revealed that m^6^A METTL3, METTL14, and WTAP levels were downregulated in PTB. Moreover, variants of METTL14 rs62328061 and WTAP rs11752345 are related to the genetic background of PTB, indicating that these m^6^A regulators play a pivotal role in PTB (33). Notably, our research combined two GEO datasets and provided new molecules containing METTL3, VIRMA, YTHDC1, and YTHDC2 perspectives on the pathological process of TB and a comprehensive analysis of 22 core m^6^A regulators in the epitranscriptomic landscape of TB. We further developed and validated two diagnostic models based on m^6^A regulator expression profiles, both of which demonstrated excellent discriminatory power between TB patients and healthy controls, highlighting their potential as novel diagnostic biomarkers. Interestingly, these results were confirmed in clinical samples. Our innovative findings revealed that the combined AUC of these m^6^A genes and Ziehl-Neelsen staining for TB was 0.953, which may reduce the missed detection rate of Ziehl-Neelsen staining in clinical settings and significantly improve the diagnostic accuracy for TB infection. The low AUCs of individual genes underscored the multifactorial futures of TB pathogenesis and supported the rationale for developing multi-gene rather than single-gene diagnostic models. As in the research of Ding (31), they highlight the potential of key m^6^A regulatory genes YTHDF1, HNRNPC, LRPPRC, and ELAVL1 as diagnostic biomarkers for TB through machine learning, which demonstrated their crucial role in TB pathogenesis. We not only revealed the importance of multiple key m^6^A molecules in the diagnosis of tuberculosis from bioinformatic analysis but also confirmed their function in clinical samples and clinical TB tests. We are the first to construct and validate a multivariate diagnostic model based on a panel of m^6^A regulators, revealing superior performance compared to individual markers and even showing additive value to traditional smear microscopy. This may move beyond associations and tangible clinical applications.

In addition to their diagnostic potential, we explored the biological implications of m^6^A modification patterns in TB. Using unsupervised clustering analysis, we identified two distinct m^6^A modification patterns among patients with TB, and each pattern was associated with different immune profiles and biological characteristics. Pattern 1 exhibited higher levels of activated immune cells such as gamma delta T cells and neutrophils, suggesting a more robust immune response. In contrast, pattern 2 was characterized by a predominance of type 1 T helper cells and a relatively milder immune response. These differences in immune microenvironment characteristics might influence the progression and treatment outcomes of TB, highlighting the need for personalized therapeutic strategies based on m^6^A modification patterns, which might offer a potential framework for advancing fundamental research into personalized therapeutic strategies. Importantly, immune subclassification has been successfully applied in the study of cancers and infectious diseases, where it has helped clarify how variations in local immune microenvironments contribute to divergent disease trajectories. In the future, we will focus on elucidating the distinct immune response profiles associated with tuberculosis patterns through detailed molecular and cellular characterizations in larger or independent cohorts. Such investigations will deepen our understanding of TB immunopathology and provide the mechanistic insights necessary for the rational design of pattern-specific interventions. Extensive research has highlighted the pivotal role of gamma delta T cells play multiple crucial roles in anti-TB immunity, as demonstrated in several studies. For instance, gamma delta T cells can produce key antimicrobial cytokines such as interferon-γ (IFN-γ) and tumor necrosis factor-α (TNF-α), which are crucial for controlling Mycobacterium tuberculosis (Mtb) infection (34, 35). γδ T cells possess cytotoxic functions and can directly kill infected cells through CD16-mediated pathways, which play a particularly prominent role in chronic tuberculosis infection (36). γδ T cells provide rapid protection in the early stages of infection, particularly in terms of mucosal immunity. They are the main circulating γδ T cell subset (Vγ2Vδ2 T cells) in humans and other primates, and are capable of quickly recognizing microbial phosphoantigens (such as HMBPP) and exerting multifunctional effects, including IL-17 production, thereby playing a key role in the early control of tuberculosis infection (37, 38). Taken together, these findings implicate m^6^A regulatory genes in the modulation of TB progression via immune and inflammatory mechanisms.

Furthermore, we derived a 22-gene signature from these m^6^A patterns and used it to classify TB patients into two novel subtypes (subtypes A and B). These subtypes, while immunologically similar, were driven by different biological processes: subtype A by heme metabolism and subtype B by TGF-β signaling, revealing a previously unrecognized layer of heterogeneity in TB. Infection with Mycobacterium tuberculosis might affect iron metabolism in the host, and since iron is a key component of heme, it may lead to anemia or oxidative stress. In addition, TGF-β plays a role in immune regulation and may promote immunosuppression in TB (39). Overall, the m^6^A -derived subtypes were linked to heme metabolism and the TGF-β signaling pathway, revealing a novel axis of TB heterogeneity independent of classic immune infiltration, suggesting that m^6^A modification influences TB pathophysiology through previously unexplored biological pathways.

Our study has several limitations. First, our validation was performed on peripheral blood, which might not fully mirror the complex m^6^A modifications and cellular interactions in the lungs during infection. our findings in PBMCs serve as a foundational discovery. In the future, validation in relevant lung-resident cells or tissues is essential to fully elucidate the localized mechanistic role of m6A modification in TB pathogenesis. The sample size for the clinical validation cohort was also moderate, potentially limiting the statistical power and generalizability of our diagnostic models. Second, although strong associations and patterns were identified, functional experiments to establish causal relationships between specific m^6^A regulators and immune or metabolic phenotypes were lacking. To illustrate these limitations, more samples, including bronchoalveolar lavage fluid (BALF) or granuloma tissues from patients with TB, will be conducted to obtain more precise data. A multicenter study with a larger cohort is required to confirm our findings. Cell culture and animal models will be used to explore the mechanisms by which m^6^A regulators influence specific immune pathways through m^6^A -dependent mechanisms.

In conclusion, our study systematically delineates the dynamic landscape of m^6^A modifications throughout the pathological progression of TB. We identified a cluster of m^6^A -regulated genes and elucidated their potential involvement in the immune microenvironment and the biological heterogeneity of TB. These results help to bridge the current gaps in TB epigenetics and highlight the promising potential of m^6^A -based biomarkers for diagnostic applications and targeted therapeutic interventions. Our results support the hypothesis that m^6^A modification serves as a critical regulatory mechanism influencing TB pathogenesis and progression, laying the foundation for subsequent research on early detection and personalized treatment strategies. This study provides valuable insights that may guide future clinical translation of TB management.

## Data Availability

The raw data supporting the conclusions of this article will be made available by the authors, without undue reservation.
